# Assessment of Health-Related Quality of Life and Biomarkers in Long COVID: A 12-Month Longitudinal Feasibility Cohort

**DOI:** 10.3390/jcm14227931

**Published:** 2025-11-08

**Authors:** Fahad Alghamdi, Robert Meertens, Abasiama Dick Obotiba, Lorna W. Harries, Sarah Appleby, Kinan Mokbel, Karen M. Knapp, William David Strain

**Affiliations:** 1 Department of Radiologic Technology, College of Applied Medical Sciences, Qassim University, Buraydah 52571, Saudi Arabia; 2 Department of Health and Care Professions, Faculty of Health and Life Sciences, University of Exeter, Exeter EX1 2LU, UK; 3 Department of Clinical and Biomedical Sciences, Faculty of Health and Life Sciences, University of Exeter, Exeter EX1 2LU, UK

**Keywords:** long COVID, bone turnover markers, joint pain, health-related quality of life, inflammatory markers

## Abstract

**Background/Objectives**: Long COVID (LC) causes persistent symptoms, including fatigue, musculoskeletal (MSK) pain, and a lower quality of life. It is hypothesised that chronic low-grade inflammation in LC could impact bone, joints, and muscle microcirculation, but evidence is limited. Our aim is to assess health-related quality of life (HRQoL) and circulating inflammation, bone turnover markers (BTM), and vitamin D in LC individuals to explore their potential association with MSK function. **Methods**: Prospective longitudinal cohort; LC *n* = 45, well-recovered (WR) *n* = 40; 12 ± 2 months follow-up. Baseline and follow-up assessments included evaluations of HRQoL and pain-rating questionnaires, and blood analysis of inflammatory and bone turnover markers (BTM). **Results**: More females were in the LC group. LC reported significantly lower HRQoL compared to WR, with no change over 12 months. LC had higher vitamin D levels at baseline, median 29.46 ng/mL (23.75; 35.06) compared to WR 20.36 ng/mL (15.995; 27.65) (*p* = 0.0021). Both groups experienced significant increases in vitamin D after 12 months: WR median from 21.4 ng/mL (16.34; 27.89) to 29.58 ng/mL (25.33; 41.74), (*p* =< 0.001) and LC median from 32.695 ng/mL (23.665; 35.1) to 35.89 ng/mL (30.1; 41.2), (*p* = 0.0023). Pain rating showed LC also experienced more hand pain at baseline median 1 (0; 5), (*p* = 0.003). There were no differences between groups in BTM or cytokines over time. **Conclusions**: This feasibility cohort showed that LC is associated with a reduction in HRQoL and joint symptoms; however, no significant changes were observed in the inflammatory markers, indicating the need for ongoing monitoring. Future studies should explore MSK, muscle function via imaging, and ways to enhance musculoskeletal health and well-being.

## 1. Introduction

The unprecedented health crisis in 2019 caused by the coronavirus disease 2019 (COVID-19) became a global pandemic, spreading across many countries, and causing respiratory illness and deaths linked to SARS-CoV-2 [1,2]. Symptoms, severity, and recovery times during COVID-19 vary, and SARS-CoV-2 infection can lead to post-acute sequelae, with most recovering in a month, but some report prolonged health issues [3]. Various names have been used to refer to this condition; however, the preferred term for this condition is “long COVID” (LC) [4].

LC can impact and elevate the risk of disorders across multiple organ systems, leading to a reduction in quality of life, with symptoms varying significantly in severity and duration [5,6,7]. With over 200 reported symptoms associated with LC, the most frequently reported are fatigue, cognitive impairment, joint pain, anxiety, and depression [5,8]. Reports highlight diverse MSK symptoms linked to LC, mainly fatigue and widespread joint and muscle pain, with many experiencing back or neck pain, complicating the condition [9,10,11,12,13,14,15,16].

Reports also indicate that patients with LC frequently exhibit vitamin D insufficiency, with deficiency associated with an increased number of symptoms [17,18,19]. LC systemic inflammation may affect the MSK system, with some studies suggesting that acute COVID-19 markers influence bone metabolism [20]. Evidence shows that LC patients have elevated inflammatory markers and cytokines [21,22]. Joint pain post-COVID-19 can resemble other inflammatory conditions, with 2% to 65% experiencing symptoms lasting one month to a year [23].

This paper aims to assess HRQoL and circulating markers of inflammation, as well as BTM and vitamin D in LC individuals. However, additional data, such as imaging, is planned as part of a broader effort to explore the underlying mechanisms in greater depth.

## 2. Materials and Methods

### 2.1. Study Design and Ethics Approval

This prospective longitudinal feasibility study received ethical approval from the Yorkshire and the Humber–Bradford Leeds Research Ethics Committee (Ref: 23/YH/0031), 12 April 2023. All participants provided written informed consent.

### 2.2. Study Population

Participants were recruited from LC clinics, ongoing studies focused on LC participants, and through advertisements using leaflets, posters, and social media platforms. A total of 85 participants were recruited from a cohort of individuals who had previously been diagnosed with COVID-19. Participants were divided into two groups: the LC group and the WR group. The inclusion/exclusion criteria included further imaging investigation (Table 1). As mentioned above, while the primary aim was to assess changes in HRQoL and blood analysis, additional imaging data were planned to explore significant LC effects in more depth. Thus, pregnant women were excluded due to potential risks associated with radiation exposure from imaging, in accordance with standard safety guidelines.

The LC group (*n* = 45) included individuals with persistent symptoms lasting ≥12 weeks after acute COVID-19, self-reported based on clinical diagnosis by a physician or self-reported symptoms aligned with NICE criteria [24]. The WR group (*n* = 40) comprised individuals who had confirmed SARS-CoV-2 infection but recovered fully without persistent symptoms. Recruitment began in June 2023. Because the date of acute infection was rarely verifiable, “time since infection” could not be determined for most participants, so it has not been considered in the analysis.

### 2.3. Data Collection and Assessments

Weight (kg) and height (cm) were measured using a calibrated Marsden M-530 bariatric scale and wall-mounted stadiometer. Body mass index (BMI) was calculated as weight (kg)/height (m^2^).

### 2.4. Blood Tests

To determine the presence of the COVID-19 virus within the bloodstream, the subgenomic RNA (sgRNA) test was used [25]. The sgRNA is a proxy for the virus with infective potential, as it is only present in an actively replicating virus. In contrast, conventional COVID-19 PCR picks up viral fragments, whether active or inactive. The latter can give false positives due to the persistence of viral fragments in the absence of an active viral reservoir. In the absence of nasal or nose swabs, this is an acceptable proxy and were used in other peer-reviewed studies [26]. The kinetics of the assay are given in the paper describing the design of the assay [25]. The limit of detection (LOD) for quantification is >100 viral copies, and the reproducibility r^2^ was 0.996.

Cytokine analysis offers insight into LC’s circulatory mechanisms, possibly involving prolonged SARS-CoV-2 presence [27]. Reports link systemic inflammation to pro-inflammatory cytokines [28,29,30], which are crucial for bone homeostasis [31]. During COVID-19’s acute phase, markers like IL-6, IFN-γ, and TNF-α affect bone mechanisms [20]. Two studies systematically found elevated CRP and cytokines/chemokines, including TNF-α, IFN-γ, and IL-6, in LC patients [21,22]. Regarding the BTM, IOF, and IFCC, they recommend using one bone formation marker (s-PINP) and one resorption marker (s-CTX), measured by standardised assays, in studies [32].

Blood samples were collected to assess inflammatory markers, BTM, vitamin D level, and SARS-CoV-2 sgRNA. To maintain the integrity of BTM, participants were required to fast for 10 h and avoid intense exercise for 48 h before the test [33]. Additionally, participants were asked to refrain from taking multivitamin supplements on the day of the appointment, as these may affect BTM levels [34,35]. Due to these requirements, blood collection was scheduled as the first procedure of the appointment.

Blood samples were obtained through a phlebotomy using a butterfly needle BD Vacutainer Safety-Lok 21 g Blood Collection Set, BD Vacutainer^®^ serum separator tube (SST)™ (Clot activator/polymer gel), and PAXgene^®^ Blood RNA Tube.

The SST tube was used for analysis of BTM (PINP ng/mL), CTX ng/mL, and vitamin D (25 OH D ng/mL) by an external laboratory, Sheffield Biochemical Laboratory, using Cobas e411, Roche Diagnostics (Mannheim, Germany) analyser. After ensuring clotting, a centrifuge at 2000× *g* for 10 min in a swinging bucket centrifuge [36] was used to isolate serum. The serum was then transferred to small aliquots in anticoagulated vacutainer bottles and stored at −80 °C.

The PINP/CTX ratio serves as a relative BTM balance indicator because it offers a broader view of bone remodelling and is more reliable than absolute PINP and CTX levels. It minimises confounding effects, such as circadian and physiological fluctuations. Research indicates that this ratio gives better insight into overall bone health in older adults than individual markers [37,38].

The second tube is the PAXgene RNA tube, filled with stabilising reagent to prevent backflow. After stabilisation, the sample is stored at −80 °C. The analysis was conducted by the Royal Devon University Healthcare NHS Foundation Trust at the Research, Innovation, Learning, and Development (RILD) building. SARS-CoV-2 (sgRNA) was tested [39], along with quantitative PCR (qPCR) inflammatory markers (CRP, IL-6, IFN-γ, and TNF-α).

#### 2.4.1. RNA Extraction

RNA was extracted from blood samples collected in PAXgene Blood RNA tubes. Blood samples were thawed for 3 h at room temperature before processing with the PAXgene Blood RNA kit (762174, Qiagen, Germany), according to the manufacturer’s instructions. RNA concentration was determined using a NanoDrop (Thermo Fisher Scientific, Waltham, MA, USA).

#### 2.4.2. cDNA Synthesis

RNA was converted to cDNA using the PrimeScript™ RT Master Mix (Perfect Real Time) kit (RR036A, Takara, Japan). Each reaction contained 1000 ng RNA, 4 µL 5X PrimeScript RT Master Mix, and sufficient RNase-Free dH2O to reach a total reaction volume of 20 µL. Reactions were mixed gently and incubated at 37 °C for 15 min, followed by 85 °C for 5 s. Samples were stored at −20 °C.

#### 2.4.3. qPCR Analysis

cDNA was diluted 1:3 in RNase-Free dH2O to ensure sufficient volume for all assays. qPCR was performed using TaqMan™ Universal Master Mix II, no UNG (4440048, Thermo Fisher Scientific, Waltham, MA, USA), and TaqMan probes (Thermo Fisher Scientific, Waltham, MA, USA). Genes of interest included COL1A1 (Hs00164004_m1), COL1A2 (Hs01028956_m1), CRP (Hs00265044_m1), IL-6 (Hs00174131_m1), IFN-γ (Hs00989291_m1), and TNF-α (Hs00174128_m1). Housekeepers included GUSB (Hs00939627_m1), HPRT (Hs02800695_m1), PPIA (Hs04194521_s1), and UBC (Hs05002522_g1). Individual qPCR reactions contained 2.5 µL TaqMan™ Universal Master Mix II, 0.25 µL TaqMan probe, 1 µL cDNA, and 1.25 µL H2O. Each sample was run in triplicate in a 384-well plate. qPCR was performed using the QuantStudio™ 12K (Thermo Fisher Scientific, Waltham, MA, USA) with the following cycling conditions: 50 °C for 2 min, 95 °C for 10 min, 40–45 cycles of 95 °C for 15 s, and 60 °C for 1 min. qPCR results were analysed using the 2^−ΔΔCt^ method.

### 2.5. Quality of Life and Joint Pain Assessment

Subjective measures of HRQoL, which provide descriptive health state profiles through the EQ-5D-5L, and joint pain assessed via the VAS, provide critical patient-reported outcomes. HRQoL was evaluated using EQ-5D-5L, which assesses mobility, self-care, usual activities, pain/discomfort, and anxiety/depression. Each of these has five levels, coded from 1 to 5, corresponding to responses (no = 1, slight = 2, moderate = 3, severe = 4, and extreme problems = 5). The second part contains a rating scale ranging from 0, indicating the worst imaginable health, to 100, indicating the best imaginable health, as a VAS [40]. EQ-5D utility index (UI) reflects average public preferences, for which the UK standard was adapted from the EUROQOL [41,42], which has a maximum value of 1 (for domains’ health state 11111), indicating the best possible health, and a minimum value of −0.594 (for 55555), indicating the worst possible health.

The EQ-5D-5L is a simple, validated screening questionnaire recommended by NICE for managing the long-term effects of COVID-19 as a control measure for confining LC symptoms and monitoring their condition [43]. Research indicates high test–retest reliability, making it appropriate for monitoring changes over time and for comparative analysis among groups [44,45]. Various studies have shown that EQ-5D-5L demonstrates good-to-excellent test–retest reliability for the index score, with substantial agreement at the dimension level. Recent online administration also reported strong agreement across dimensions [46,47].

Joint pain severity was recorded using a VAS. It indicates the presence of pain in the hand or knee joint, along with its intensity. The scale ranged from 0 to 10, which correlates with visual and verbal scales as follows: 0 indicates no pain, 1–3 indicates mild pain, 4–6 denotes moderate pain, and 7–10 signifies severe pain and its impact on daily activities [48]. Studies have demonstrated excellent test–retest reliability in musculoskeletal populations, such as osteoarthritic knee pain, with an ICC of approximately 0.97, and have also shown good reliability, with ICC values exceeding 0.80, across an inflammatory rheumatic cohort [49,50].

### 2.6. Statistical Analysis

Data were analysed using Stata v18.0 (StataCorp, College Station, TX, USA). Descriptive statistics, including means and standard deviations (SD), were used for normally distributed continuous variables. Categorical variables were presented as counts and percentages. Associations between categorical variables were assessed as appropriate using the chi-squared test when the groups were independent, provided that the expected value in at least 80% of the cells was ≥5, or Fisher’s exact test was used when more than 20% of cells had expected frequencies < 5 [51,52,53].

Given the exploratory and feasible nature of this study, as well as the small sample size, statistical analyses were limited to independent *t*-tests (or Mann–Whitney U tests for non-parametric data) for between-group comparisons and Wilcoxon signed-rank tests for within-group changes over 12 months. Only completed records were used. While this method allowed for the initial investigation of differences, it does not fully account for repeated measures or interactions between groups and time.

All tests were two-tailed, and a *p*-value cutoff of <0.01 was considered statistically significant for essential differences. Statistical significance was set at *p* < 0.01 beforehand, a more stringent threshold than the usual *p* < 0.05, based on advice from a consulting statistician to reduce the likelihood of type I errors due to multiple univariate comparisons. No formal correction for multiple comparisons raises potential biases and limitations; however, the analyses were mainly exploratory and aimed to identify patterns for future research. Nevertheless, the risk of false-positive results from multiple testing persists, and all findings should be interpreted with this in mind. When relevant, effect sizes and 95% confidence intervals are also provided to offer insights into the magnitude and reliability of the observed differences.

## 3. Results

### 3.1. Demographics and Characteristics

The full characteristics of the participants are summarised in Table 2. A total of 85 participants were included, comprising 45 in the LC group and 40 in the WR group. The average age was 52.22 ± 9.94 years for the LC group and 51 ± 15.2 years for the WR group, showing no significant differences in age between the groups (*p* = 0.658). The LC group had a significantly higher percentage of female participants compared to the WR group (84.45% vs. 47%, *p* < 0.001). Most participants’ ethnicity was White (94.12%), with a small proportion of individuals from Indian, Pakistani, Black African, and Chinese backgrounds.

There were no significant differences in BMI between the LC and WR groups (*p* = 0.2142). However, both groups were similar in terms of BMI, socio-economic status, smoking, and daily alcohol consumption, as shown in Table 2. At baseline, 17.8% of LC groups and 7.5% of WR participants reported using hormonal replacement therapy (HRT). Regarding vitamin D supplements, 38% of participants in the LC group reported taking supplements, compared to 20% in the WR group (*p* = 0.096).

**Figure 1:**
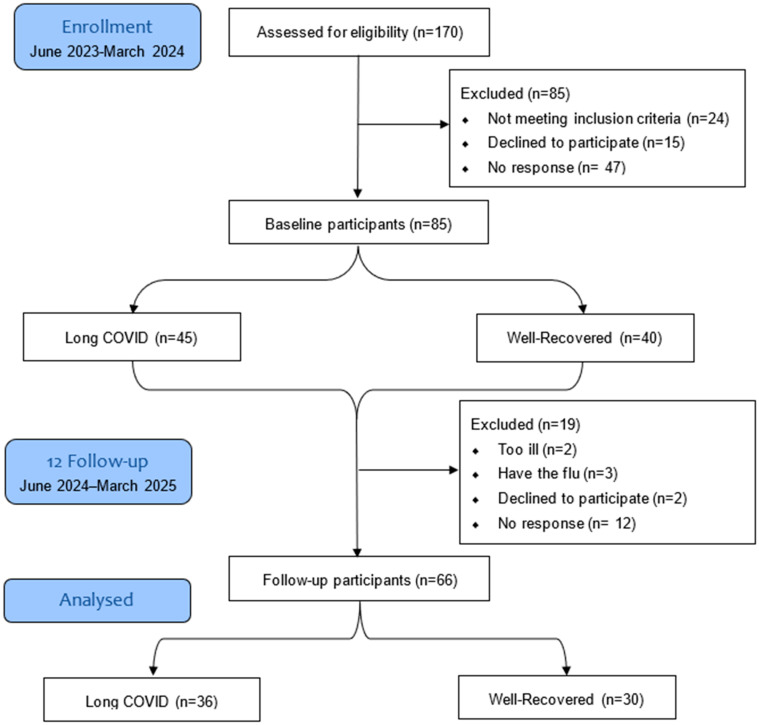
Flow chart of recruitment numbers at each study phase. The overall dropout in the 12-month period was 22.4%.

Of the 85 participants who completed the baseline assessment, 19 (22.4%) were lost to follow-up. Of these, 12 (63%) did not respond to the follow-up invitation, 5 (26.3%) were too ill due to ongoing disease, and 2 (10.5%) were not interested in continuing with the study (Figure 1). At follow-up, conducted 12 ± 2 months later, 66 participants were re-evaluated. Of these, 44 (66.67%) were female (LC = 29 vs. WR = 15), *p* = 0.017. The mean age was similar across the groups (*p* = 0.8928). LC still had an insignificantly higher BMI at follow-up compared to WR (*p* = 0.09). Over 12 months, the median BMI in the LC group increased from 27.6 kg/m^2^ (IQR 24.7–33.5) to 28.8 kg/m^2^ (23.8–34.45), but this change was not statistically significant (*p* = 0.037) vs. WR (*p* = 0.52).

Socioeconomic levels were similar across groups (*p* = 0.450). The ethnic makeup of the LC group comprises 91.66% White, 2.78% Indian, 2.78% Pakistani, and 2.78% Chinese individuals. In comparison, the WR group is entirely composed of Whites. Smoking and alcohol status show no significant differences (*p* = 0.74 and *p* = 0.396), respectively.

After the baseline visit, five WR and one LC of the participants started taking medication for bone health.

### 3.2. Health-Related Quality of Life (HRQoL)

LC participants consistently reported significantly lower HRQoL across all EQ-5D-5L domains and rated their overall health significantly lower on VAS compared to WR individuals at both baseline and follow-up (*p* < 0.01). The median UI for the LC group was significantly lower than the WR group at the baseline LC 0.725 (IQR 0.55; 0.81) vs. WR 1 (IQR 0.922; 1); 95% CI −0.564; −0.415, (*p* < 0.001) and at follow-up LC 0.697 (IQR 0.53; 0.809) vs. WR 1 (IQR 0.937; 1); 95% CI −0.584; −0.452, (*p* < 0.001)] (Table 3).

**Figure 2:**
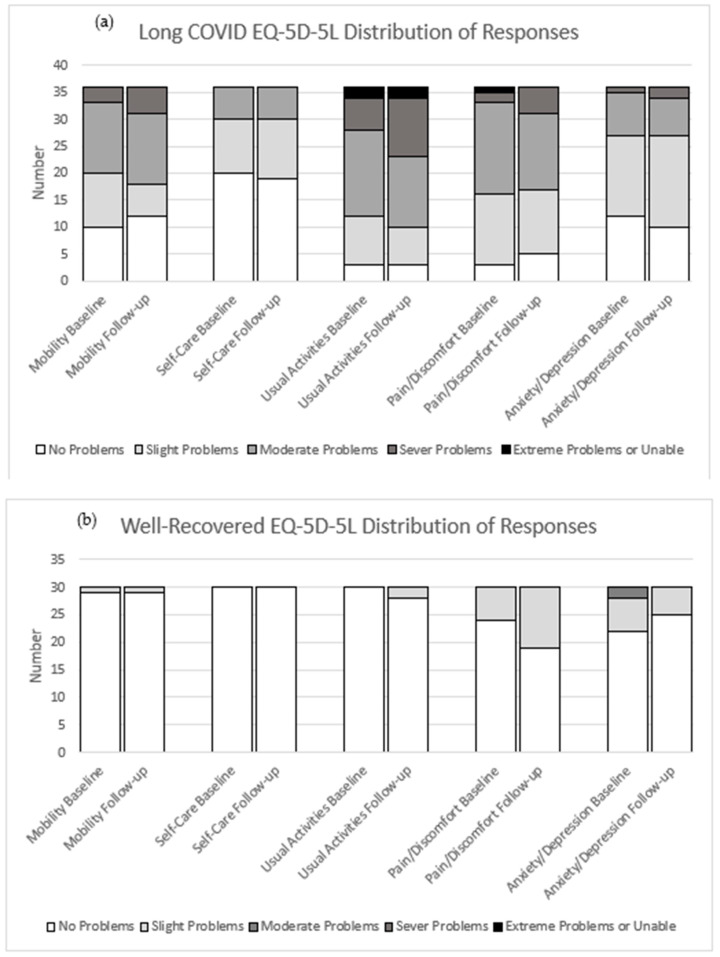
Distribution of responses to the descriptive system of the EQ-5D-5L for within-group completed records during the study period. (**a**) long COVID group (*n* = 36) and (**b**) well-recovered group (*n* = 30) at baseline and follow-up for each domain, from which the utility index is derived.

**Figure 3:**
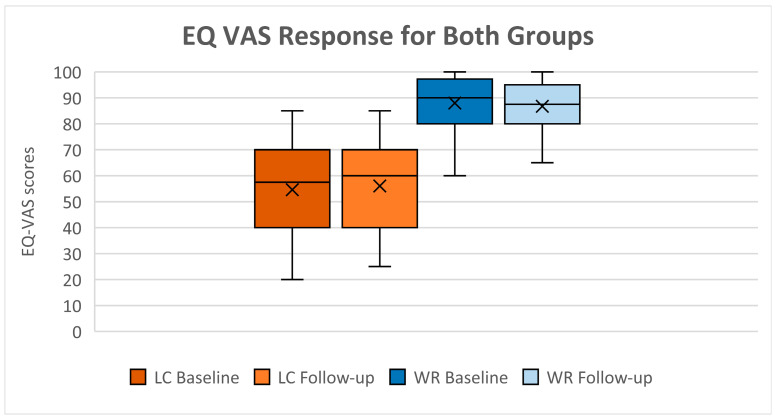
EQ VAS: visual analogue scale response for both groups; WR: Well-recovered (*n* = 30); LC: long COVID (*n* = 36).

Over 12 months, there was no significant change in HRQoL domains within the LC group. The median UI for the LC group remained low, from 0.725 (IQR 0.587; 0.809) at baseline to 0.697 (IQR 0.53–0.81) at follow-up; 95% CI −0.057; 0.024, (*p* = 0.398), whereas the WR group scored near-perfectly through the 12 months (Table 4). Response distribution of completed participants’ records across domains largely remained the same from baseline to follow-up (Figure 2a,b). However, LC reports of severe difficulties in usual activities increased from 16.7% to 30.6%. Furthermore, LC participants showed a slight 2% increase in VAS scores, whereas WR participants experienced a 2% decrease over this period (Figure 3).

### 3.3. Joint Pain

Notably, at baseline, the median hand joint pain was significantly higher in the LC group compared to the WR group, with 1 (IQR 0; 5) vs. 0 (IQR 0; 0); 95% CI 0.208; 0.526, (*p* = 0.003). However, in the follow-up, there was no significant difference in hand joint pain between the groups, with LC median 0.5 (IQR 0; 4) vs. WR median 0 (IQR 0; 0); 95% CI 0.244; 0.571, (*p* = 0.039). Additionally, the LC group reported a marginally higher pain level than the WR group at each timepoint, at baseline median 3 (IQR 0; 6) vs. 0 (IQR 0; 2); 95% CI for change 0.176; 0.434, (*p* = 0.024), at follow-up showed similar medians and IQR, with a weaker, non-significant difference (95% CI 0.087; 0.411, (*p* = 0.093)), as summarised in Table 3. Within-group comparisons reveal that participants in the LC group continued to face persistent hand and knee joint pain after 12 months, with a median hand pain level from 0.5 (IQR 0; 5) to 0.5 (IQR 0; 4); 95% CI −0.782; 0.393, (*p* = 0.624), and from 3 (IQR 0; 5) to 3 (IQR 0; 6); 95% CI −1.164; 0.553, (*p* = 0.573) knee pain level, as summarised in Table 4.

**Figure 4:**
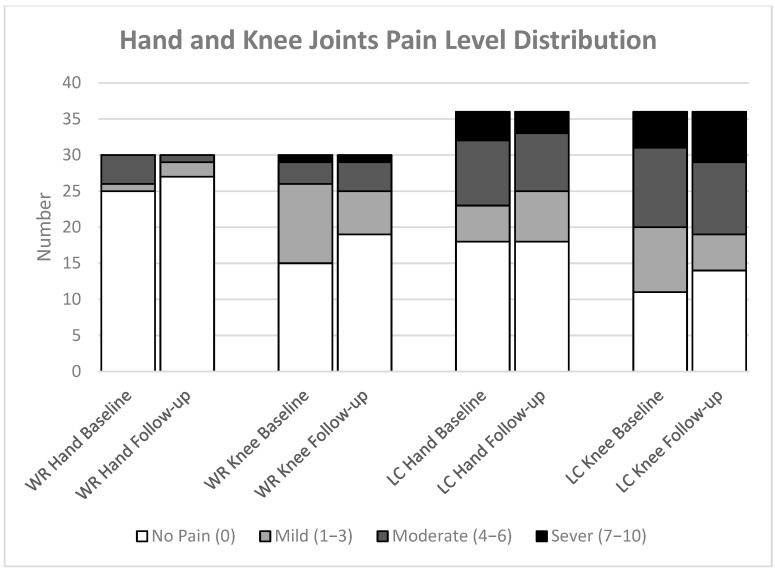
The Figure illustrates the frequency of different pain levels in the hand and knee for both groups: Well-recovered (WR) and long COVID (LC). The groups consist of WR (*n* = 30) and LC (*n* = 36).

Participants in the LC group consistently reported higher levels of joint pain in the hand and knee compared to those in the WR group at each timepoint. At baseline, participants reported 11% hand and 14% knee severe pain, while 8% and 19% reported severe pain during the follow-up, respectively, as shown in Figure 4.

**Figure 5:**
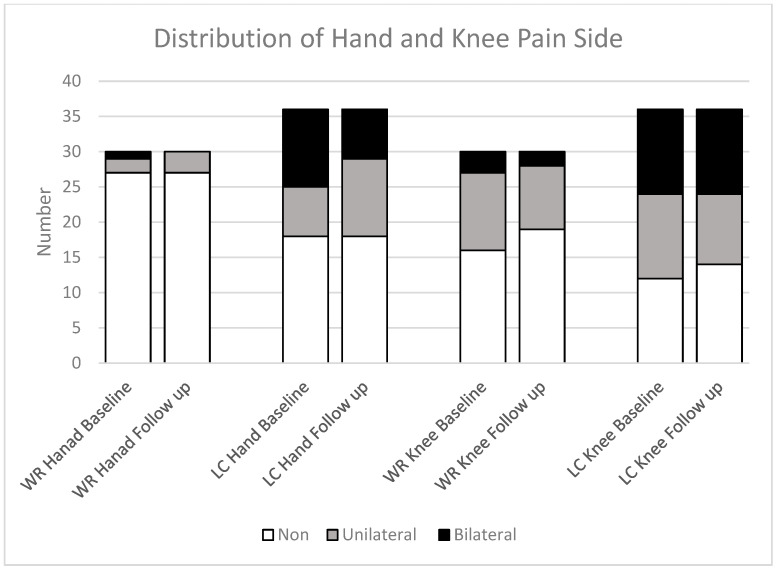
Side-joint pain in the hand and knee distribution among completed study groups; LC: long COVID; WR: Well-recovered (WR: *n* = 30; LC: *n* = 36). Pain is categorised as absent (non), unilateral (one side), or bilateral (both sides).

The distribution of hand and knee pain among completed participant data at baseline shows that the LC group had a higher prevalence of pain in the bilateral hand (31%), with 19% experiencing unilateral pain. Knee joint pain was present in 33% of participants, both bilateral and unilateral, at baseline, with similar rates observed at follow-up. The WR group experienced less hand pain, with 3% having bilateral pain and 7% having unilateral pain, with no bilateral cases at follow-up and a slight increase to 19% in unilateral pain. Unilateral knee joint pain decreased from 37% to 30%, and unilateral pain decreased from 10% to 7% (Figure 5).

### 3.4. Blood Analysis

The blood analysis assessed SARS-CoV-2 RNA, cytokines, BTMs, and vitamin D. Neither the LC nor the WR groups had detectable SARS-CoV-2 sgRNA, and CRP levels were too low to be measured reliably. Cytokines were quantified at the level of the mRNA transcript by RTqPCR and expressed as a ratio of CRP relative to the average of a set of three housekeeping genes. Individual crossing points were in the range of 36.1 to 37.5 for 10 replicates, with the remaining 455 replicates reading no crossing point, meaning that no transcript was detected. In comparison, the crossing points of the housekeeping genes were 23.9–29.3, 26.5–31.0, and 20.6–25.8 out of a total of 45 cycles. qPCR is a well-accepted means of quantifying gene expression.

In addition, sensitivity analyses were conducted to evaluate how hormone replacement therapy (HRT) might affect the BTM ratio. The findings showed that HRT had no significant effect on the BTM ratio in both the overall cohort and the LC group (Supplementary S1). Similarly, additional analyses investigating the impact of supplementation on serum 25(OH)D levels found no significant relationship, indicating that supplementation did not alter vitamin D levels in this cohort or specifically within the LC group (Supplementary S2).

Cytokine levels at each study point showed no statistically significant differences between the two groups, as summarised in Table 3. Notably, LC participants showed lower baseline levels of TNF-α compared to WR participants, but this difference did not reach the predetermined level of significance (LC median 0.771, IQR 0.605–0.983 vs. WR 1.028, IQR 0.678–1.284; 95% CI (−0.0276; −0.032), *p* = 0.0151). By follow-up, the difference was no longer observed (*p* = 0.1628). Gender stratification revealed that males primarily contributed to the overall reduction: LC males had a median TNF-α of 0.7 (IQR 0.53–0.86), compared to 1.12 (IQR 0.88–1.28) in WR males, with an insignificant difference (*p* = 0.0182). For females, the difference was less pronounced, with LC females at a median of 0.8 (IQR 0.61–0.98) and WR females at 0.94 (IQR 0.66–1.28; *p* = 0.18). Within-group comparisons showed no significant changes in TNF-α, IL-6, or IFN-γ levels from baseline to follow-up in either group (Table 4).

Results of BTM ratio comparison between LC and WR groups at the baseline and follow-up are summarised in Table 3. At both assessment points, there were no statistically significant differences in the PINP/CTX ratio between the two groups. Over the course of 12 months, within-group comparisons revealed an increase in the BTM ratio in both the LC and WR groups. The increase was more pronounced in the LC group than the WR group; however, the difference was not significant between the two groups. The median for the LC group increased from 149.2 (IQR: 113.2–190.1) to 169.4 (IQR: 143.6–206.2), 95% CI (3.583; 31.629), (*p* = 0.0111), while in the WR group it increased from 143.2 (IQR: 124.2–182.6) to 168.151 (IQR: 132.6–194.7), 95% CI (8.227; 38.819), (*p* = 0.192) (Table 4).

Vitamin D comparison between the LC and WR groups at the baseline and follow-up showed that vitamin D levels differed significantly at baseline, with the LC group exhibiting a higher median 25 OH D concentration of 29.46 ng/mL (IQR: 23.75–35.06) compared to 20.36 ng/mL (IQR: 15.99–27.65) in the WR group, 95% CI (0.072; 0.318), (*p* = 0.0021). By follow-up, the difference in vitamin D levels between the groups was no longer statistically significant (*p* = 0.099). Within-group analyses revealed significant increases in vitamin D levels over 12 months in both groups: from 32.70 ng/mL (IQR: 23.66; 35.1) to 35.89 ng/mL (IQR: 30.1; 41.2), 95% CI (2.752; 9.259), (*p* = 0.0023) in LC, and from 21.36 ng/mL (IQR: 16.34; 27.89) to 29.58 ng/mL (IQR: 25.33; 41.74), 95% CI (4.889; 11.603), (*p* = 0.0001) in WR (Table 4).

## 4. Discussion

LC is often a debilitating condition that affects at least 10% of SARS-CoV-2 infections. This study aims to evaluate HRQoL and circulating markers of inflammation, as well as BTM and vitamin D in LC individuals. The study found significant differences in two strands: between the two groups, LC had reduced HRQoL and reported more pain in the hand joints. Additionally, they had, on average, a higher level of vitamin D at baseline compared to the WR group. Within 12 months, there was an improvement in vitamin D levels for both groups. Furthermore, no significant differences were observed in BTM ratio or inflammatory markers. This suggests that factors beyond visible inflammation, such as deconditioning, metabolic changes, autonomic dysfunction, or central pain mechanisms, might contribute to ongoing symptoms. Notably, the study offers a 12-month longitudinal perspective, which is scarce in the current literature, and was identified as a gap by our previous systematic review [54].

### 4.1. Long COVID Associated with Deterioration of Health-Related Quality of Life

LC participants reported a significantly lower HRQoL than WR, with the most significant deficits in domains related to physical functioning, fatigue, and pain. The median utility index within the LC group was significantly lower (0.78 and 0.697) than the perfect score of WR individuals at both baseline and one year later, indicating a considerable perceived health discrepancy. These findings align with the more exhaustive LC research, indicating ongoing, fluctuating symptoms that hinder daily activities and participation. The continued presence of these deficits over a year, despite mostly normal biomarker results, suggests that subjective symptom burden may not always be accounted for by noticeable inflammation that the chosen tools can detect.

This substantiates prior findings indicating that LC exerts a persistent physical impact and contributes to the scant available long-term data concerning recovery trajectories within LC populations. While the short-term effects of COVID-19 adversely affect mental health, physical health may experience recovery [55]. On the contrary, LC significantly influences physical health but does not exacerbate mental health issues beyond the levels observed during the pandemic. Consistent with previous studies [56,57,58,59], individuals with LC reported notably diminished HRQoL, particularly in domains related to mobility and pain. Research shows that physical fatigue with weakness and pain in the muscle in LC is more debilitating than cognitive fatigue, which is similar to other conditions such as fibromyalgia and multiple sclerosis [60]. In LC, it has been associated with structural and functional connectivity in frontal, temporal, and cerebellar regions [61].

### 4.2. No Association of Bone Turnover Markers in Long COVID

No significant differences were observed between LC and WR in BTMs, with no notable decline within either group over the one year. This indicates that, in this cohort, LC did not lead to detectable disrupted bone remodelling during the study period. Our systematic review concluded that COVID-19 (both acute and post-acute) may adversely affect bone health [54]. It noted increases in bone regulatory markers, reductions in bone formation and resorption, and lower BMD. Some studies have found that non-human models infected with SARS-CoV-2 experience acute harm to trabecular bone, resulting in alterations in bone structure and an increase in osteoclast numbers [62,63,64,65]. While this study did not find a significant difference in BTM between LC and WR individuals, the observed trend of lower values in LC participants merits further exploration in future research. The proposed mechanisms encompass viral persistence, or reactivation, induction of autoimmunity, tissue damage, and the development of microclots [66].

It is known that changes in bone volume occur over time, after the acute infection has resolved, whereas BTMs can change during the active phase of the disease. The results did not reveal any significant differences in BTM between participants with LC and WR throughout the study period. This indicates that approximately one year following infection, LC is not associated with markedly abnormal ongoing bone resorption or formation. A decreased level of circulating osteocalcin (OC) had been observed in critically ill ICU COVID-19 patients in comparison to non-COVID-19 patients [67]. Additionally, individuals with non-severe COVID-19 had lower levels of total P1NP and osteocalcin N-terminal in the middle (N-MID OC) compared to healthy individuals [68]. Another study found that the COVID-19 patient group had a lower level of serum OPG compared to the matched control group [65].

### 4.3. No Association of Inflammatory Markers in Long COVID

No viral persistence was observed in the study population. Additionally, the inflammatory marker profiles within the LC cohort showed no statistically significant differences when compared to the WR group. Specifically, CRP and other commonly measured inflammatory markers primarily remained within normal ranges and demonstrated a similar pattern across both groups. These findings do not indicate a notable viral persistence or elevation in inflammation within the LC cohort.

This finding is somewhat unexpected, as previous studies have identified residual inflammation in LC cases. The discrepancy may be due to differences in cohort characteristics or timing; the participants were evaluated at a median of less than two years post-infection, whereas many had experienced symptoms for over two years.

Conversely, systematic review reports that elevated cytokine levels are frequently concentrated in patients in the early stages or those exhibiting more severe LC symptoms [21]. This systematic review compared cytokines in LC versus recovered, healthy controls, and often in those with active infection; symptom subgroups were also analysed. Key findings were higher in IL-6 and TNF-α (plus CRP), with broader panels (e.g., chemokines, IFNs, and IL-17) differing versus controls and symptom clusters. Sampling windows post-infection varied: <3 months (28.6%), 3–6 months (32.1%), ≥6 months (10.7%), mixed (3.6%), or unspecified (25%), with most cytokines measured after symptoms appeared, limiting prognostic conclusions. Yet, our findings are consistent with recent observations, indicating that systemic inflammation may decrease in numerous LC patients within one year or, at the very least, may not reach levels that differentiate them from fully WR individuals [69,70].

Other explanations focus on ongoing changes in the MSK in LC patients, which can lead to reduced HRQoL, even when inflammatory markers appear normal. Recent research suggests that patients with LC may experience mitochondrial and endothelial dysfunction in skeletal muscles, capillary loss, and post-exertional myopathy, sometimes accompanied by amyloid-like deposits that worsen after activity [71,72]. Additionally, cardiovascular autonomic problems, such as decreased heart rate variability and chronotropic incompetence, can further restrict physical activity despite normal inflammatory biomarkers like CRP or ESR levels [73,74]. Overall, these findings imply that fatigue in LC is mainly driven by neuromuscular and autonomic dysfunction rather than ongoing systemic inflammation [75,76,77].

### 4.4. Vitamin D Level Initially High in Long COVID and Improved in Both Groups During 12 Months

Interestingly, vitamin D levels in both groups increased significantly over the 12 months, possibly due to supplementation or seasonal influences. LC participants may have proactively begun supplements more frequently following their illness, possibly being influenced by advice or personal choice, which could have elevated their vitamin D levels. Meanwhile, WR started with lower baseline vitamin D levels, with a median of ~21 ng/mL, and “caught up” by the follow-up. Participants and their clinicians took action during the year. Vitamin D exhibits immunomodulatory characteristics that help regulate the immune system. It plays a crucial role in adjusting both the adaptive and innate immune systems by influencing cytokines and cell signalling pathways [78,79]. Studies have linked its deficiency to dysregulated immune responses and heightened vulnerability to respiratory infections [80].

### 4.5. Long COVID Linked to Persistent Joint Pain Within 12 Months

Joint pain scores remained elevated in LC participants, with no resolution observed after one year. This was an unexpected observation, likely resulting from incidental or transient findings in individuals who were otherwise recovered. It is acknowledged that viral infections may affect joints, including hepatitis B or C, Epstein–Barr virus, and HIV, among others [81,82,83,84]. It had been suggested that the widespread joint and muscle pain is likely due to acute COVID-19 [85]. Similarly, half of the patients, 180 days post SARS-CoV-1, with considerable joint pain in multiple joints, were observed with a negative MRI scan, leading to the assumption of neurogenic pain or low-grade synovitis that could not be detected via MRI, lasting up to 4 years [86].

There are two theories regarding the relationship between COVID-19 and articular manifestations. One suggests that COVID-19, accompanied by viremia or a cytokine storm, may cause viral arthritis, or it may be a non-specific consequence of the cytokine storm associated with symptomatic forms of the disease. However, no confirmed cases have been reported so far [87]. Another theory suggests that arthritis may be triggered by an inflammatory response to the systemic condition caused by COVID-19, which may lead to reactive arthritis [88].

This longitudinal study found that individuals with LC showed a consistent decline in HRQoL and ongoing joint pain over 12 months, despite normal inflammatory profiles and stable bone turnover markers. Baseline vitamin D was higher in LC and increased in both groups, likely due to supplementation or seasonal effects. Mechanisms beyond inflammation or bone remodelling, such as deconditioning, autonomic dysregulation, metabolic issues, or central pain mechanisms, may drive persistent symptoms. The findings support multidisciplinary care focused on pain management or functional restoration. Future research should involve larger cohorts with longer follow-up, mechanistic studies using advanced assessments, and targeted interventions, such as rehabilitation. These efforts aim to refine personalised care and improve outcomes for people with LC. For LC patients with declining HRQoL, a multidisciplinary rehabilitation approach is advised. This includes personalised, graded physical activity with pacing to prevent post-exertional symptoms, in addition to physiotherapy, psychological support, and comorbidity management. Evidence also supports adding autonomic rehab, nutritional optimisation, and gradual return-to-work plans to enhance function and reduce fatigue [89,90]. Regular monitoring of musculoskeletal health, instead of only inflammatory markers, is crucial for guiding recovery and avoiding relapse.

### 4.6. Limitations

The study has a few limitations worth noting. Internal validity is affected by the statistical approach, using paired and independent tests, because it ignores repeated measures and interactions. Repeated measures or linear mixed models are better for capturing longitudinal effects and handling missing data. This study conducted many univariate statistical tests without using formal corrections for multiple comparisons, raising the risk of false positives, although a more conservative *p* < 0.01 threshold was used instead. Future research should consider implementing formal multiplicity adjustments or focusing analyses on pre-specified hypotheses. Causal inferences are also inherently limited by the observational cohort design, where confounding factors may influence the results. For example, any observed improvements or declines might be due to natural recovery or external factors rather than being directly caused by LC. Some participants started new treatments, including osteoporosis medications and vitamin D, during the study, which may have influenced BTM. The increase in vitamin D levels, likely due to supplementation, may have improved bone outcomes and masked differences attributed to other LC factors. The potential impact of seasonal recruitment could have influenced the vitamin D levels between groups at baseline. However, most baseline assessments occurred in winter. Additionally, vitamin D’s seasonality means baseline differences and within-year changes in 25(OH)D might reflect timing effects rather than true group differences, without adjusting for calendar month or supplementation. We also lacked standardised data on analgesic use and physical activity, which could affect pain scores and bone markers. External validity is reduced, as most participants were from semi-urban areas in the Southwest UK, and primarily Caucasian, with females being more dominant in the LC group. Thus, findings may not generalise to more diverse populations and could over-represent females. Participation was also voluntary, which may have led to more motivated individuals with more severe symptoms joining and participating in follow-up, potentially introducing recruitment bias. Approximately 22% of participants dropped out during the year, potentially causing attrition bias, which should be considered in future studies.

## 5. Conclusions

We evaluated HRQoL, joint pain scores, serum vitamin D, BTM, and standard inflammatory markers in LC participants. LC was associated with poorer HRQoL, particularly in physical health domains, and with greater baseline hand joint pain, suggesting that physical therapy should focus on symptom-driven reconditioning and rehabilitation, raising important questions regarding the physiological mechanisms underpinning the observed changes in HRQoL. We found no differences between the LC and WR groups concerning inflammatory markers or bone profiles, underscoring the promise of imaging-derived phenotyping as a complementary or alternative approach to blood-based markers. This highlights the need for further research into the MSK health of individuals with LC, as MSK sequelae may not be readily captured through blood markers alone, potentially requiring imaging data to unravel the mechanisms underpinning the reduced HRQoL in LC.

## Figures and Tables

**Table 1:** Study inclusion/exclusion criteria.

Inclusion Criteria	Exclusion Criteria
Adults aged ≥18 years of any gender and ethnicity.	Participants who had hospitalisation due to COVID-19 requiring intubation, ICU admission, or ventilatory support (to exclude post-intensive care syndrome).
WR participants with a history of SARS-CoV-2 infection confirmed via RT-PCR or antigen testing.	Individuals with pre-existing osteoporosis or metabolic bone diseases (e.g., primary hyperparathyroidism, osteogenesis imperfecta).
LC participants met the WHO and NICE definitions of long COVID or had a confirmed diagnosis of LC.	Those undergoing long-term corticosteroid therapy (≥5 mg prednisolone daily) or taking bisphosphonates, denosumab, or teriparatide.
	Pregnant or breastfeeding women, due to the use of ionising radiation in DXA scans.
	Participants with recent fractures (<12 months) or conditions affecting joint health, such as rheumatoid arthritis (RA) or systemic lupus erythematosus (SLE).

RT-PCR: reverse transcription polymerase chain reaction; WHO: World Health Organisation; NICE: National Institute for Health and Care Excellence; LC: long COVID; WR: Well-recovered.

**Table 2:** BMI: Body mass index (kg/m^2^); yr: years; WR: Well-recovered; LC: long COVID; (*n*): participants number at each timepoint; *p*-values using appropriate tests (Mann–Whitney U or independent *t*-tests) for continued data based on data normality and X^2^ or Fisher’s exact test for categorical data; data are presented as median with interquartile range (IQR), mean ± standard deviation (SD), or *n* (%): number and percentages as appropriate; (₽): *t*-tests; (‡): Mann–Whitney U; (¥): Fisher’s exact test; (X): X^2^ test; * Statistically significant at *p* < 0.01.

Participant Characteristics								
Variables	Baseline				Follow-up			
	WR (*n* = 40)	LC (*n* = 45)	Test Value	*p*	WR (*n* = 30)	LC (*n* = 36)	Test Value	*p*
Age (yr) ^(₽)^	51 ± 15.17	52.22 ± 9.94	t (83) = −0.444	0.658	52.83 ± 14.85	53.28 ± 10.08	t (64) = −0.144	0.885
Gender (Female), *n* (%) ^(X)^	19 (47)	38 (84.45)	X^2^ (1) = 13.084	<0.001 *	15 (50)	29 (80.56)	X^2^ (1) = 6.875	0.009 *
BMI (kg/m^2^) ^(‡)^	26.6 (23.8; 30.65)	27.9 (24.7; 33)	z = −1.242	0.214	25.55 (23.4; 29.3)	28.8 (23.8; 34.45)	z = −1.707	0.087
Ethnicity, *n* (%) ^(¥)^				0.459				1.00
White or not stated	39 (97.5)	41 (91.1)			30 (100)	33 (91.7)		
Indian	0 (0.0)	2 (4.4)			0 (0.0)	1 (2.8)		
Pakistani	0 (0.0)	1 (2.2)			0 (0.0)	1 (2.8)		
Black African	1 (2.5)	0 (0.0)			0 (0.0)	0 (0.0)		
Chinese	0 (0.0)	1 (2.22)			0 (0.0)	1 (2.8)		
Socio-economic, *n* (%) ^(X)^			X^2^ (2) = 2.649	0.266			X^2^ (2) = 1.602	0.449
Upper	20 (50)	23 (51.1)			15 (50)	18 (50)		
Upper Middle	13 (32.5)	19 (42.2)			12 (40)	17 (47.2)		
Lower Middle	7 (17.5)	3 (6.7)			3 (10)	1 (1.8)		
Smoking status, *n* (%) ^(¥)^				0.298				0.742
Non-smoker	26 (65)	35 (77.8)			21 (70)	26 (72.2)		
Ex-smoker	8 (20)	8 (17.8)			4 (13.3)	6 (16.7)		
Light smoker (less than 10)	2 (5)	2 (4.44)			2 (6.67)	3 (8.3)		
Moderate smoker (10 to 19)	3 (7.5)	0 (0.0)			1 (3.33)	1 (2.8)		
Heavy smoker (20 or over)	1 (2.5)	0 (0.0)			2 (6.67)	0 (0.0)		
Alcohol status, *n* (%) ^(¥)^				0.526				0.396
Non	15 (37.5)	22 (48.89)			16 (53.33)	21 (58.3)		
<1 unit per day	12 (11.3)	10 (22.2)			7 (23.33)	9 (25)		
1–2 units per day	9 (22.5)	8 (17.8)			4 (13.33)	4 (11.1)		
3–6 units per day	1 (2.5)	4 (8.9)			3 (10)	0 (0.0)		
7–9 units per day	1 (2.5)	0 (0.0)			0 (0.0)	1 (2.8)		
>9 units per day	2 (5)	1 (2.22)			0 (0.0)	1 (2.8)		
Hormonal Replacement Therapy, *n* (%) ^(X)^	3 (7.5)	8 (17.8)	X^2^ (1) = 1.985	0.159				
Supplementation of Vitamin D, *n* (%) ^(X)^	6 (20)	14 (38.9)	X^2^ (1) = 3.054	0.080				
Bone Health Medication, *n* (%) ^(X)^	-	-		-	5 (16.7)	1 (2.8)	X^2^ (1) = 1.985	0.084

**Table 3:** UI: Utility Index; WR: Well-recovered; LC: long COVID; VAS: visual analogue scale; TNF-α: Tumour Necrosis Factor alpha; IL-6: Interleukin-6; IFN-γ: interferon gamma; CTX: C-terminal telopeptide of type I collagen; PINP: procollagen type I N-propeptide; 25 OH D: 25-hydroxyvitamin D; (*n*): participants’ number at each timepoint; CI: confidence interval; *p*-values using appropriate tests (Mann–Whitney) for continuous data based on data normality X^2^ test for categorical data; All variables are ordinal or continuous and non-normally distributed; values are reported as median (interquartile range: IQR); (‡): Mann–Whitney U; (¥): Fisher’s exact test; (X): X^2^ test; * Statistically significant at *p* < 0.01.

Systemic Results Compared Between the LC and WR Groups at Baseline and Follow-Up													
	Variables	*n*	Baseline					*n*	Follow-up				
			WR	LC	Test Value	*p*	95% CI		WR	LC	Test Value	*p*	95% CI
(EQ-5D-5L)	Mobility ^(X)^	40/45	0 (0; 0)	0.058 (0; 0.076)	X^2^ (3) = 38.995	<0.001 *		30/36	0 (0; 0)	0.067 (0; 0.076)	X^2^ (3) = 28.308	<0.001 *	
	Self-Care ^(X)^		0 (0; 0)	0 (0; 0.05)	X^2^ (2) = 20.465	<0.001 *			0 (0; 0)	0 (0; 0.05)	X^2^ (2) = 19.081	<0.001 *	
	Usual Activities ^(X)^		0 (0; 0)	0.063 (0.05; 0.063)	X^2^ (4) = 59.792	<0.001 *			0 (0; 0)	0.063 (0.05; 0.162)	X^2^ (4) = 48.796	<0.001 *	
	Pain/Discomfort ^(X)^		0 (0; 0.032)	0.084 (0.063; 0.084)	X^2^ (4) = 42.561	<0.001 *			0 (0; 0.063)	0.084 (0.063; 0.084)	X^2^ (3) = 26.886	<0.001 *	
	Anxiety/Depression ^(X)^		0 (0; 0.078)	0.075 (0; 0.104)	X^2^ (3) = 17.111	0.001 *			0 (0; 0)	0.078 (0; 0.091)	X^2^ (3) = 18.172	<0.001 *	
	UI ^(‡)^		1 (0.922; 1)	0.72 (0.55; 0.808)	z = 7.041	<0.001 *	(−0.564; −0.415)		1 (0.937; 1)	0.697 (0.53; 0.809)	z = 6.632	<0.001 *	(−0.584; −0.452)
	VAS ^(‡)^		87.5 (80; 95)	55 (40; 70)	z = 6.993	<0.001 *	(−0.519; −0.423)		90 (80; 97)	60 (40; 70)	z = 6.362	<0.001 *	(−0.532; −0.430)
Joint Pain	Hand Pain ^(¥)^		0 (0; 0)	1 (0; 5)		0.003 *	(0.208; 0.526)		0(0; 0)	0.5 (0; 4)		0.039	(0.244; 0.571)
	Knee Pain ^(¥)^		0 (0; 2)	3 (0; 6)		0.024	(0.176; 0.434)		0 (0; 2)	3 (0; 6)		0.093	(0.087; 0.411)
Biomarkers	TNF-α ^(‡)^	40/45	1.03 (0.68; 1.28)	0.77 (0.61; 0.98)	z = 2.430	0.015	(−0.0276; −0.032)	29/35	0.96 (0.68; 1.39)	0.86 (0.76; 0.98)	z = 1.396	0.162	(−0.258; 0.053)
	IL-6 ^(‡)^		1.03 (0.63; 1.41)	1.08 (0.72; 1.66)	z = −0.977	0.328	(−0.063; 0.0187)		0.81 (0.48; 1.35)	1.06 (0.62; 1.44)	z = −0.816	0.414	(−0.085; 0.205)
	IFN-γ ^(‡)^		1.03 (0.55; 1.53)	0.73 (0.52; 1.44)	z = 1.083	0.278	(−0.0195;0.057)		0.82 (0.48; 1.23)	0.95 (0.51; 1.40)	z = −0.573	0.566	(−0.103; 0.188)
	PINP/CTX ratio ^(‡)^	40/44	150.6 (124.5; 184.6)	152.91 (125; 200.3)	z = −0.448	0.654	(−0.098; 0.156)	30/36	168.2 (132.6; 194.7)	171.5 (144.6; 208.9)	z = −0.541	0.588	(−0.106; 0.1844)
	25 OH D ng/mL ^(‡)^	40/45	20.36 (15.995; 27.65)	29.46 (23.75; 35.06)	z = −3.073	0.0021 *	(0.072; 0.318)	30/36	29.58 (25.33; 41.74)	35.89 (30.095; 41.16)	z = −1.648	0.099	(−0.028; 0.267)

**Table 4:** UI: Utility Index; WR: Well-recovered; LC: long COVID; EQ VAS: visual analogue scale; TNF-α: Tumour Necrosis Factor alpha; IL-6: Interleukin-6; IFN-γ: Interferon gamma; (*n*): participants number at both timepoints; CI: confidence interval; *p*-values based on Wilcoxon signed-rank; All variables are ordinal or continuous and non-normally distributed; values are reported as median (interquartile range: IQR) at baseline and follow-up; * Statistically significant at *p* < 0.01.

Paired Analysis Within Groups from Baseline to Follow-Up											
	Variables		WR (*n* = 30)					LC (*n* = 36)			
		*n*	Baseline	Follow-Up	CI	*p*	*n*	Baseline	Follow-Up	CI	*p*
(EQ-5D-5L)	Mobility	30	0 (0; 0)	0 (0; 0)		1.000	36	0.058 (0; 0.076)	0.067 (0; 0.076)		0.528
	Self-Care		0 (0; 0)	0 (0; 0)		1.000		0 (0; 0.05)	0 (0; 0.05)		0.948
	Usual Activities		0 (0; 0)	0 (0; 0)		0.157		0.063 (0.05; 0.063)	0.063 (0.05; 0.162)		0.060
	Pain/Discomfort		0 (0; 0)	0 (0; 0.063)		0.058		0.084 (0.063; 0.084)	0.084 (0.063; 0.084)		0.867
	Anxiety/Depression		0 (0; 0.078)	0 (0; 0)		0.282		0.078 (0; 0.091)	0.078 (0; 0.091)		0.362
	UI		1 (0.922; 1)	1 (0.937; 1)	−0.028; 0.019	0.946		0.725 (0.587; 0.809)	0.697 (0.53; 0.809)	−0.057; 0.024	0.398
	VAS		87.5 (80; 95)	90 (80; 97)	−1.133; 3.666	0.264		57.5 (40; 70)	60 (40; 70)	−5.653; 8.486	0.868
Joint Pain	Hand Pain		0 (0; 0)	0 (0; 0)	−0.122; 0.755	0.917		0.5 (0; 5)	0.5 (0; 4)	−0.782; 0.393	0.624
	Knee Pain		0.5 (0; 2)	0 (0; 2)	−0.805; 1.005	0.371		3 (0; 5)	3 (0; 6)	−1.164; 0.553	0.573
Biomarkers	TNF-α	29	0.98 (0.67; 1.28)	0.96 (0.68; 1.39)	−0.122; 0.262	0.991	35	0.77 (0.61; 0.98)	0.86 (0.76; 0.98)	−0.107; 0.133	0.412
	IL-6		0.85 (0.58; 1.35)	0.81 (0.48; 1.35)	−0.248; 0.190	0.566		1.25 (0.72; 1.88)	1.06 (0.62; 1.44)	−0.489; 0.012	0.076
	IFN-γ		0.91 (0.43; 1.52)	0.82 (0.48; 1.23)	−0.862; 0.210	0.112		0.84 (0.55; 1.63)	0.95 (0.51; 1.4)	−0.444; 0.210	0.831
	PINP/CTX ratio	30	143.2 (124.2; 182.6)	168.151 (132.6; 194.7)	−8.227; 38.819	0.192		149.2 (113.2; 190.1)	169.4 (143.6; 206.2)	3.583; 31.629	0.011
	25 OH D ng/mL	30	21.4 (16.34; 27.89)	29.58 (25.33; 41.74)	4.889; 11.603	<0.001 *	36	32.695 (23.665; 35.1)	35.89 (30.1; 41.2)	2.752; 9.259	0.0023 *

## Data Availability

The original contributions presented in the study are included in the article (and Supplementary Material), further inquiries can be directed to the corresponding author.

## Supplementary Materials

The following supporting information can be downloaded at: https://www.mdpi.com/article/10.3390/jcm14227931/s1. Supplementary S1: Regression analysis of the use of Hormone Replacement Therapy (HRT) effects on PINP/CTX Ratio (ng/mL) and Bone Mineral Density (g/cm^2^) in the Overall Cohort and Long COVID (LC) Subgroup. Supplementary S2: Regression analysis of the impact of the supplementation on 25 OH D ng/ml levels.

## Abbreviations

The following abbreviations are used in this manuscript:

BTM	Bone turnover markers
CTX	C-Terminal Telopeptide of Type 1 Collagen
EQ-5D-5L	EuroQol 5-Dimension 5-Level
HRQoL	Health-related quality of life
HRT	Hormone replacement therapy
IL	Interleukins
INF-γ	Interferon-Gamma
MSK	Musculoskeletal
PINP	Procollagen Type I N Propeptide
TNF-α	Tumour Necrosis Factor-Alpha
VAS	Visual analogue scale
