# Supplementary material for: Assessment of Health-Related Quality of Life and Biomarkers in Long COVID: A 12-Month Longitudinal Feasibility Cohort
Source: J Clin Med. 2025 Nov 8;14(22):7931. doi: 10.3390/jcm14227931 (PMC12653226; doi:10.3390/jcm14227931)
Supplement: Supplementary file 1 [file jcm-14-07931-s001.zip › jcm-3948056-supplementary.pdf]

## Supplementary Materials

**Supplementary S1**: Regression analysis of the use of Hormone Replacement Therapy (HRT) effects on PINP/CTX Ratio (ng/ml) and Bone Mineral Density (g/cm²) in the Overall Cohort and Long COVID (LC) Subgroup.

| | HRT on the Overall Cohort | | | | HRT on Long COVID Subgroup | | | |
|---|---|---|---|---|---|---|---|---|
| | *n* | ß (95% CI) | $R^2$ | *p* | *n* | ß (95% CI) | $R^2$ | *p* |
| PINP/CTX ratio | 84 | 20.98 (-15.38; 57.33) | 0.016 | 0.25 | 44 | 36.39 (-11.58, 84.36) | 0.053 | 0.13 |
| L1-L4 | 65 | -0.094 ( -0.232;0.044) | 0.028 | 0.18 | 33 | -0.0182 (-0.161;0.125) | 0.002 | 0.8 |
| Total Body | 84 | -0.044 (-0.12;0.03) | 0.02 | 0.23 | 45 | -0.005 (-0.084;0.74) | 0.0004 | 0.9 |
| RT Femoral Neck | 84 | -0.069 (-0.157;0.019) | 0.029 | 0.123 | 45 | -0.032 (-0.138;0.074) | 0.009 | 0.54 |
| LT Femoral Neck | 84 | -0.045 (-0.143;0.053) | 0.01 | 0.37 | 45 | 0.022 ( -0.099;0.142) | 0.003 | 0.72 |
| RT Total Hip | 84 | -0.078 (-0.172;0.02) | 0.032 | 0.1 | 45 | -0.038 (-0.148;0.07) | 0.012 | 0.48 |
| LT Total Hip | 83 | -0.054 (-0.153;0.044) | 0.015 | 0.28 | 44 | 0.01 ( -0.098;0.12) | 0.001 | 0.85 |

Supplementary S1: BTM: Bone Turnover Markers; BMD: bone mineral density (g/cm2); L1-L4: lumbar spine; Rt: Right; Lt: Left; (n): participants number; CI (95% Confidence Interval); β: beta coefficient; $R^2$: coefficient of determination; p-values from linear regression models; * Statistically significant at $p<0.01$.

**Supplementary S2:** Regression analysis of the impact of the supplementation on 25 OH D ng/ml levels.

| | Supplementation on the Overall Cohort | | | | Supplementation on Long COVID Subgroup | | | |
|---|---|---|---|---|---|---|---|---|
| | n | ß (95% CI) | R2 | *p* | n | ß (95% CI) | R2 | *p* |
| 25 OH D | 85 | 4.43 (-1.25; 10.11) | 0.028 | 0.12 | 45 | 3.05(-3.37; 9.48) | 0.021 | 0.34 |

Supplementary S2: 25 OH D: 25-hydroxyvitamin D; (n): participants number; CI (95% Confidence Interval); β: beta coefficient; $R^2$: coefficient of determination; p-values from linear regression models; * Statistically significant at $p<0.01$.
